# Automated Speech Markers of Alzheimer Dementia: Test of Cross-Linguistic Generalizability

**DOI:** 10.2196/74200

**Published:** 2025-10-15

**Authors:** Paula Andrea Pérez-Toro, Franco J Ferrante, Gonzalo Pérez, Boon Lead Tee, Jessica de Leon, Elmar Nöth, Maria Schuster, Andreas Maier, Andrea Slachevsky, Maria Luisa Gorno-Tempini, Agustín Ibáñez, Juan Rafael Orozco-Arroyave, Adolfo García

**Affiliations:** 1 Pattern Recognition Lab Friedrich-Alexander-Universität Erlangen-Nürnberg Erlangen Germany; 2 GITA Lab Facultad de Ingeniería Universidad de Antioquia Medellín Colombia; 3 Massachusetts General Hospital Boston, MA United States; 4 Centro de Neurociencias Cognitivas University of San Andrés Buenos Aires Argentina; 5 Consejo Nacional de Investigaciones Científicas y Técnicas Ciudad Autónoma de Buenos Aires Argentina; 6 Facultad de Ingeniería University of Buenos Aires Ciudad Autónoma de Buenos Aires Argentina; 7 Global Brain Health Institute University of California San Francisco, CA United States; 8 Memory and Aging Center Department of Neurology University of California, San Francisco San Francisco, CA United States; 9 Trinity College Dublin Dublin Ireland; 10 Department of Otorhinolaryngology Ludwig-Maximilians-Universität München Munich Germany; 11 Neuropsychology and Clinical Neuroscience Laboratory (LANNEC) Faculty of Medicine University of Chile Santiago Chile; 12 Geroscience Center for Brain Health and Metabolism (GERO) Faculty of Medicine University of Chile Santiago Chile; 13 Memory and Neuropsychiatric Clinic (CMYN) Neurology Department Hospital del Salvador Santiago Chile; 14 Servicio de Neurología Departamento de Medicina Clínica Alemana Santiago Chile; 15 Latin American Brain Health (BrainLat) Institute Adolfo Ibáñez University Santiago Chile; 16 Departamento de Lingüística y Literatura Facultad de Humanidades Universidad de Santiago de Chile Santiago Chile

**Keywords:** Alzheimer disease, digital biomarkers, automated speech and language analysis, interpretability, cross-linguistic validity

## Abstract

**Background:**

Automated speech and language analysis (ASLA) is gaining momentum as a noninvasive, affordable, and scalable approach for the early detection of Alzheimer disease (AD). Nevertheless, the literature presents 2 notable limitations. First, many studies use computationally derived features that lack clinical interpretability. Second, a significant proportion of ASLA studies have been conducted exclusively in English speakers. These shortcomings reduce the utility and generalizability of existing findings.

**Objective:**

To address these gaps, we investigated whether interpretable linguistic features can reliably identify AD both within and across language boundaries, focusing on English- and Spanish-speaking patients and healthy controls (HCs).

**Methods:**

We analyzed speech recordings from 211 participants, encompassing 117 English speakers (58 patients with AD and 59 HCs) and 94 Spanish speakers (47 patients with AD and 47 HCs). Participants completed a validated picture description task from the Boston Diagnostic Aphasia Examination, eliciting natural speech under controlled conditions. Recordings were preprocessed and transcribed before extracting (1) speech timing features (eg, pause duration, speech segment ratios, and voice rate) and (2) lexico-semantic features (lexical category ratios, semantic granularity, and semantic variability). Machine learning classifiers were trained with data from English-speaking patients and HCs, and then tested (1) in a within-language setting (with English-speaking patients and HCs) and (2) in a between-language setting (with Spanish-speaking patients and HCs). Additionally, the features were used to predict cognitive functioning as measured by the Mini-Mental State Examination (MMSE).

**Results:**

In the within-language condition, combined speech timing and lexico-semantic features yielded maximal classification (area under the receiver operating characteristic curve [AUC]=0.88), outperforming single-feature models (AUC=0.79 for timing features; AUC=0.80 for lexico-semantic features). Timing features showed the strongest MMSE prediction (R=0.43, *P*<.001). In the between-language condition, speech timing features generalized well to Spanish speakers (AUC=0.75) and predicted Spanish-speaking patients’ MMSE scores (R=0.39, *P*<.001). Lexico-semantic features showed lower performance (AUC=0.64) and no significant MMSE prediction (R=–0.31, *P*=.05). The combined model did not improve results (AUC=0.65; R=0.04, *P*=.79).

**Conclusions:**

These results suggest that while both timing and lexico-semantic features are informative within the same language, only speech timing features demonstrate consistent performance across languages. By focusing on clinically interpretable features, this approach supports the development of clinically usable ASLA tools.

## Introduction

Alzheimer disease (AD) is a neurodegenerative condition involving an insidious decline of semantic and episodic memory alongside other functions [1]. Its current prevalence, of 55 million, will likely triple by 2050, with cases increasing by 116% in high-income countries and 250% in low- or middle-income countries [2-4]. This underscores the need for noninvasive, scalable markers that facilitate disease detection and monitoring [5]. Automated speech and language analysis (ASLA) meets these requisites [6-8].

In ASLA studies, patients and healthy controls (HCs) are simply required to speak, be it through spontaneous (eg, memory description), semispontaneous (eg, picture description), or nonspontaneous (eg, paragraph reading) tasks [9]. Thereupon, their recordings and transcripts can be digitally analyzed to identify disease-sensitive features [10,11]. This approach has been leveraged to detect early-stage cases [6,8,12], support differential diagnosis [7], predict dementia onset [6], and capture cognitive decline [13] and brain atrophy patterns [14,15]. As ASLA also reduces testing time and costs, it represents a promising framework to foster global equity in dementia assessments [16]. However, such potential remains unmet, as most studies target features with low interpretability and tests of between-language generalizability are incipient [16,17]. This casts doubts on the approach’s clinical utility and cross-linguistic validity.

Interpretability is often undermined by 2 analytical strategies. One involves targeting heterogeneous feature sets that mix domains known to be affected (eg, semantics) and often spared (eg, morphosyntax) in AD [8,18,19]. The other relies on black-box models (eg, transformers) operating on hidden layers without clinical significance—eg, deep learning models such as BERT (Bidirectional Encoder Representations from Transformers) or Wav2vec (Meta), which produce high-dimensional representations that do not correspond to well-defined linguistic or acoustic constructs [20,21]. Both strategies yield variable outcomes that cannot be readily integrated with core clinical knowledge about AD, reducing the framework’s translational potential.

Moreover, over 40% of ASLA research on AD targets English speakers [22]. This is highly inequitable, as English is spoken by less than 20% of the world’s population [16] and AD is most prevalent in non-Anglophone countries [2-5]. Importantly, several language domains can be affected by this disease in English speakers and spared in users of other languages [22]. Thus, not all ASLA results from English-speaking cohorts may generalize well to other language groups.

Promisingly, recent developments allow the circumvention of both issues. Robust, interpretable results have been obtained in ASLA studies targeting (1) speech timing features (eg, pause duration) as proxies of lexical search effort [23-26]; and (2) lexico-semantic features, including word class features (proportion of different lexical categories) [27-29], semantic granularity (conceptual precision when naming entities) [7,30], and semantic variability (conceptual distance across successive words) [30]. Such features can reveal distinct processing demands and strategies in AD, potentially yielding maximal results when combined than when framed in isolation [8,31].

Also, these features can be tested for cross-cultural validity by training classifiers with data from English-speaking participants and testing them on users of a different language, as recently proposed [22] and preliminarily attempted in promising studies and challenges [32-34]. In particular, a 0-shot approach, involving no cross-linguistic calibration or transfer, creates highly stringent, unbiased conditions for this examination. Conceivably, cross-linguistic generalizability could be higher for speech timing features (as word retrieval effort should increase in AD irrespective of the language) than for lexico-semantic features (which vary widely between English and other languages) [35,36]. As proposed elsewhere, such features are interpretable because they reflect dysfunctions of semantic memory, a core system affected in early disease stages. This allows results to be understood in relation to well-established neuropsychological models of AD [4,5].

Against this background, we examined whether interpretable ASLA features yield cross-linguistic AD markers. Using speech timing and lexico-semantic features from a validated task [17,19,24], we first trained classifiers with English-speaking patients and HCs, and then tested them in a within-language and a between-language setting (with English and Spanish speakers as testing folds, respectively). In each case, we ran separate classifiers for timing features, lexico-semantic features, and their combination. Finally, we explored whether the most sensitive features in each setting could predict patients’ cognitive decline, indexed through the Mini-Mental State Examination (MMSE) [37]. Relative to current literature [38-40], our approach is unique in its integration of clinically motivated acoustic and linguistic features with unimodal and fusion-based classifiers for 0-shot within- and between-language classification and severity prediction. Adding novelty, our design involves Latino individuals, a large, underserved population with a high prevalence of AD [41,42].

For the within-language setting, we hypothesized that interpretable features would enable patient identification in both modalities, with the best outcomes resulting from their combination. For the between-language setting, we anticipated better cross-linguistic generalization for timing than for lexico-semantic features. Finally, considering recent findings [43,44], we anticipated that the most discriminatory features in each setting would reliably predict patients’ MMSE scores. By testing these hypotheses, we seek to further ASLA research on AD with a translational, cross-cultural ethos.

## Methods

### Study Design

**Figure 1:**
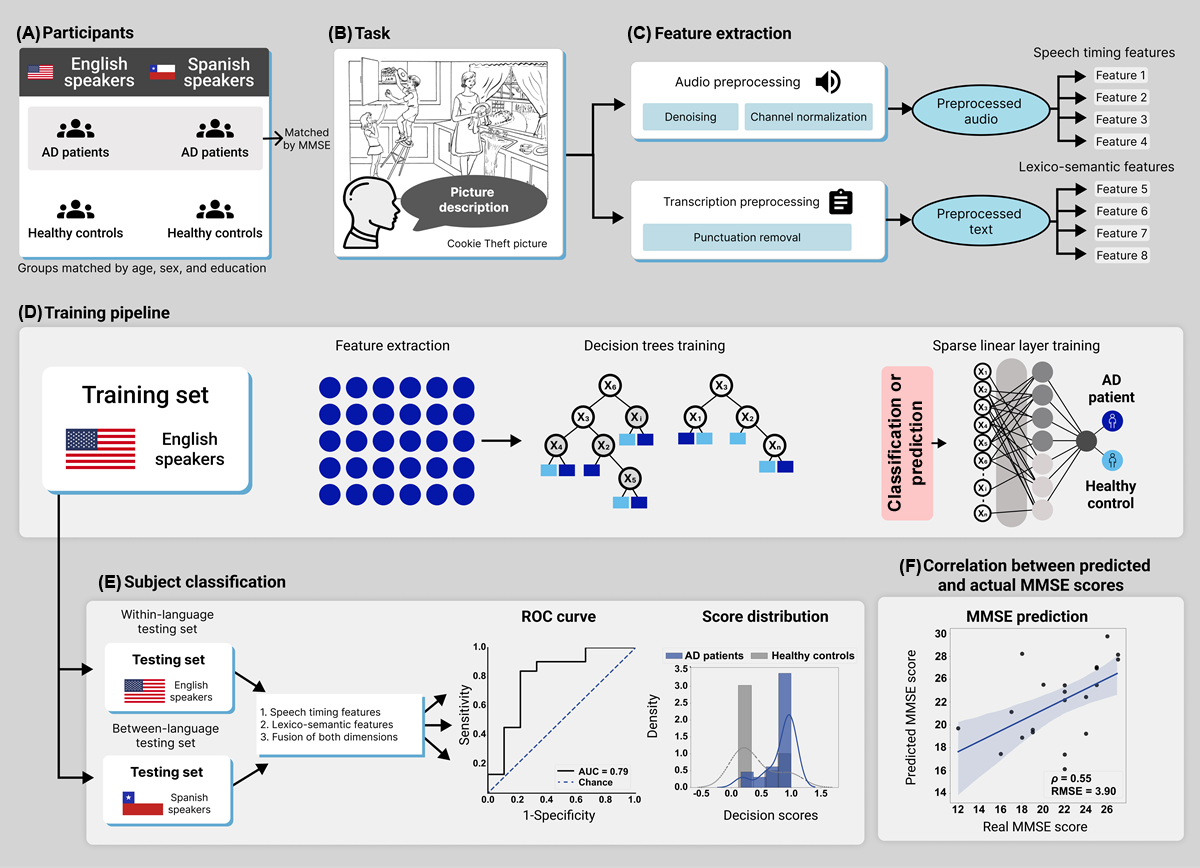
Study design. (A) This study involved English- and Spanish-speaking patients with AD and HCs, matched for sociodemographic variables (with patient groups also matched for MMSE scores). (B) Participants were recorded as they described the Cookie Theft picture. (C) Audios and transcripts were preprocessed with standard procedures before the extraction of speech timing and lexico-semantic features. (D) A machine learning classifier was trained with data from English-speaking patients with AD and HCs through a pipeline with decision trees and sparse linear models. (E) Classification performance was assessed in a within-language setting (with English-speaking patients and HCs) and in a between-language setting (with Spanish-speaking patients and HCs), considering 1=speech timing features, 2=lexico-semantic features, and 3=their combination via early fusion. The results were visualized via ROC curves and score distribution plots. (F) We computed correlations between the patients’ actual MMSE scores and those predicted by each feature set. AD: Alzheimer disease; AUC: area under the curve; HC: healthy control; MMSE: Mini-Mental State Examination; RMSE: root-mean-squared error; ROC: receiver operating characteristic.

Our study involved an opportunistic design. Preregistration was not feasible as the protocols’ stimuli, prompts, speech recording settings, and accompanying measures were established before the present investigation was conceived. Still, the analysis plan was established in the National Institutes of Health–approved project R01AG075775 (aim 1c), led by the corresponding author. Moreover, the current study extends and refines a previously reported analytical strategy [32], focused on embeddings as opposed to interpretable features. The current study’s methods are diagrammed in Figure 1.

### Participants

This study used a cross-sectional design based on a convenience sample. The initial datasets comprised 242 native English speakers for the English dataset and 198 native Spanish speakers for the Spanish dataset. Native English speaker data came from the Pitt corpus [45], a widely used resource in ASLA research on AD [17], enabling global challenges such as Alzheimer’s Dementia Recognition Through Spontaneous Speech and its variations [1]. Native Spanish speakers belonged to a Chilean cohort at the Hospital del Salvador’s Memory and Neuropsychiatry Clinic [7,30]. Fifty-six participants were removed due to faulty recordings or incomplete metadata. Participants from both datasets were selected using custom-made Python code to ensure demographic similarity for subsequent analyses (Figure 1A), leading to the removal of an additional 173 participants. The final sample involved 211 participants across both datasets, all without any missing data. The English-language dataset (n=117), for the within-language setting, included 58 patients with AD and 59 HCs. The Spanish-language dataset (n=94), for the between-language setting, encompassed 47 patients with AD and 47 HCs [46].

All English-speaking participants completed a comprehensive neuropsychiatric evaluation, a semistructured psychiatric interview, and a neuropsychological assessment [47,48]. As the Pitt corpus’s protocol began before the establishment of NINCDS-ADRDA (National Institute of Neurological and Communicative Disorders and Stroke and the Alzheimer’s Disease and Related Disorders Association) [49] and *DSM* (*Diagnostic and Statistical Manual of Mental Disorders*) [50] criteria, inclusion in the patient group was determined by frank evidence of progressive cognitive and functional decline alongside abnormal MMSE scores (sample’s mean 19.98, SD 4.79). Use of this dataset was strategic to (1) enable direct comparisons between our results and literature benchmarks while (2) guaranteeing that the same task was used in both languages. With regards to the Spanish-language dataset, AD diagnoses were made by expert neurologists following updated, validated procedures based on NINCDS-ADRDA criteria [22,51]. Outcomes on the MMSE [37] revealed abnormal cognitive scores (mean 22.11, SD 3.87) [52]. The HC group in both datasets consisted of cognitively preserved, functionally independent individuals. No participant had a history of (other) neurological disorders, psychiatric conditions, primary language deficits, or substance abuse, and all of them had normal or corrected-to-normal vision and hearing.

All 4 groups were matched for sex, age, and years of education, and dialect was consistent across participants in each language group (Table 1). MMSE scores were not matched between English- and Spanish-speaking patients with AD as dementia cutoffs differ between such populations [53]. Clinical and cognitive measures for the English and the Spanish datasets were administered following formal procedures of the University of Pittsburgh School of Medicine and harmonized protocols of the Multi-Partner Consortium to Expand Dementia Research in Latin America, respectively. The feasibility and acceptability of the Spanish data overall protocol are verified by its continuous successful application in multiple studies and populations [7,10,30,54-58] (see below for details). Participants provided written informed consent under the Declaration of Helsinki. The current study was approved by the institutional ethics committee.

**Table 1:** Participants’ demographic and neuropsychological profiles.

			English speakers				Spanish speakers					Statistics					Pairwise comparisons				
			Patient with AD^a^ (N=58)		Healthy controls (N=59)		Patients with AD (N=47)		Healthy controls (N=47)		Value			*P* value		Groups			Estimate		*P* value
**Demographic data**																					
	Sex (female:male)	35:23		36:23		24:23		33:14		χ^2^=3.62			.31^b^		N/A^c^			N/A		N/A	
	Age, mean (SD)	65.21 (6.90)		66.12 (7.08)		70.19 (6.93)		64.32 (17.48)		*F*=5.68			.02^d^								
															EN^e^ AD vs EN HCs^f^			–0.48		.97^g^	
															ES AD vs ES^h^ HCs			2.78		.06^g^	
															EN AD vs ES AD			–2.47		.11^g^	
															EN HCs vs ES HCs			0.90		.85^g^	
	Years of education, mean (SD)	13.79 (2.32)		13.46 (2.01)		13.31 (4.42)		14.53 (3.96)		*F*=3.02			.10^d^		N/A			N/A		N/A	
**Neuropsychological data**																					
	MMSE^i^, mean (SD)	19.98 (4.79)		N/A		22.11 (3.87)		N/A		N/A			N/A		EN AD vs ES AD			–3.15		.02^f^	

^a^AD: Alzheimer disease.

^b^*P* values calculated via the chi-squared test.

^c^N/A: not applicable.

^d^*P* values calculated via independent measures ANOVA, with results showing the interaction between condition (patients and controls) and language (English and Spanish).

^e^EN: English.

^f^HC: healthy control.

^g^*P* values for pairwise comparisons of significant interaction effects were calculated via independent measures ANOVA with Scheffé correction; crucially, no pairwise comparison was significant despite the significant interaction for age.

^h^ES: Spanish.

^i^MMSE: Mini-Mental State Examination.

### Experimental Task

Speech was elicited through the Cookie Theft picture task (Figure 1B) from the Boston Diagnostic Aphasia Examination [59], as described in previous reports of both datasets [7,17,30,32,43,46,60,61]. The stimulus is a black-and-white drawing of a mother and her 2 children performing several actions in a kitchen. Participants were asked to view the scene and describe its events and elements in as much detail as possible, in whichever order they preferred. No time limit was imposed. Examiners intervened only if participants needed assistance or stopped talking without mentioning central details. Importantly, this picture has proven useful in capturing speech and language abnormalities in English-speaking [62,63] and Spanish-speaking [30,58,64] cohorts.

The English recordings were captured in .mp3 format at a sample rate of 44,100 Hz and 16-bit resolution, following a comprehensive protocol overseen by the Alzheimer and Related Dementias Study at the University of Pittsburgh School of Medicine [45]. The Spanish recordings were obtained in a quiet environment using laptop computers fitted with noise-canceling microphones [30]. These recordings were captured in .wav format at a sample rate of 44,100 Hz and 16-bit resolution, all managed through Cool Edit Pro 2.0.

### Data Preprocessing and Feature Extraction

#### Overview

Audio recordings and their transcripts were preprocessed and subjected to validated feature extraction procedures, as detailed below (Figure 1C).

#### Audio Preprocessing and Extraction of Speech Timing Features

Recordings were preprocessed following a validated pipeline [65]. Segments including the examiner’s voice were manually removed from the English and the Spanish recordings. The participants’ speech signals were converted to .wav format and then resampled at a rate of 16 kHz through SoX (Sound Exchange) [66]. The output was then denoised using a recurrent neural network model with complex linear coding [67], and manual inspection confirmed the absence of speech artifacts. Microphone-related biases were eliminated via channel and mean cepstral normalization [65].

Speech timing features were extracted at the whole-recording and the single-word level (Tables 2 and 3). Whole-recording features were calculated for the following variables: pause ratio, duration ratio, speech segment ratio, speech segment duration ratio, and voice ratio. Duration information was derived from an energy-based voice activity detection (VAD) algorithm to detect the presence of human speech and differentiate it from silence segments (indicating pauses), following these steps: (1) denoising using a recurrent neural network filter [67], (2) amplitude normalization, (3) VAD, and (4) segmentation and feature extraction. This pipeline follows validated procedures reported in prior literature [68]. To this end, the log energy is calculated on 25-ms windows with a 10-ms hop size, the DC (mean value of a signal) level of the energy is subtracted, and the signal is then smoothed by convolving it with a Gaussian window of 10-ms [69]. Speech segments were detected by identifying frames with log-energy levels above a heuristically defined (yet systematic) threshold by analyzing the average signal energy in silent and speech-labeled segments. In contrast, pauses were determined by frames with log-energy levels below the threshold [70]. To calculate voice rate, we identified voice segments as those whose fundamental frequency differed from 0 [65] and computed the number of voiced segments per second. We note that the primary features used in our analysis are timing-based (eg, pause duration and speech rate), which are minimally affected by MP3 compression artifacts [71]. Indeed, MP3 compression on suprasegmental acoustic measures (eg, fundamental frequency, pitch range, and level) shows minimal measurement errors, typically below 2%, on compression bitrates in the 56-320 kbps range [72]. Although spectral distortions introduced by compression may influence certain acoustic features, our preprocessing pipeline includes noise-reduction and normalization steps that help mitigate these effects [67]. Importantly, denoising and energy-based pause detection improve performance metrics such as word error rate and character error rate [73], indicating that such preprocessing enhances reliability without artificially distorting pause-related features. Word-level features consisted of (normalized) word duration and (normalized) word count. Audio segments and their corresponding transcription units were time-aligned [74] as in previous works [75,76]. The single-word features were computed taking into account (1) syllables, (2) all words, (3) stop words, and (4) content words separately, as well as (5) all possible combinations thereof.

**Table 2:** List of speech timing features.

Whole-recording level	Single-word level
Pause duration ratio	N/A^a^
Speech segment duration ratio	Word duration
Pause ratio	Normalized word duration
Pause duration	Word count
Speech segment duration	Normalized word count
Voiced rate	N/A

^a^N/A: not applicable.

**Table 3:** List of vocabulary selection features.

Word-class features	Semantic features
Noun ratio	N/A^a^
Verb ratio	Granularity
Adjective ratio	Semantic variability
Adverb ratio	N/A

^a^N/A: not applicable.

All whole-recording and word-level features were expressed in seconds, and when possible, represented in terms of 6 statistical values (mean, SD, skewness, kurtosis, minimum value, and maximal value), as in previous research [65].

#### Transcript Preprocessing and Extraction of Vocabulary Selection Features

We used previously reported transcriptions from both datasets. English recordings were transcribed using CHAT (Codes for the Human Analysis of Transcripts), a standardized system developed by the CHILDES (Child Language Data Exchange System) project [77] for transcribing and analyzing spoken language data. It uses structured tiers to represent participant information, transcribed speech, translations, and annotations. Special characters used by this procedure (eg, ?, –, (), +) were automatically removed from each text. Spanish recordings were transcribed via an automatic speech-to-text service [78] and manually revised. The rare occurrences of unintelligible words were discarded. In all cases, examiners’ interventions were removed (including the silent segment following each intervention). Transcriptions were analyzed regarding word-class and semantic features.

First, word-class features were calculated as the ratio of each content word type: nouns, verbs, adjectives, and adverbs—namely, words which, unlike functional categories, involve representational content and thus tap on core conceptual processes affected by AD [79]. We calculated these proportions relative to the text’s overall word count and the number of content words only. Second, we calculated 2 types of semantic features. We used the Natural Language Toolkit library in Python to estimate each word’s granularity using WordNet, a hierarchical graph whose nodes branch out from the highest hypernym *entity* to more specific concepts (such as *animal*, *dog*, and *bulldog*, in increasing order of granularity). Granularity is calculated as the smaller number of nodes between a word and the root node *entity*. For instance, words in bin-3 are closer to the “entity” than those in bin-10, indicating that the former refers to more general concepts [7]. We extracted distributional statistics and the proportion of words with low, intermediate, and high granularity over the total number of words. Third, in line with reported procedures [30], semantic variability was established across: (1) content words, (2) not-repeated adjacent words, and (3) not-repeated adjacent content words. We allocated individual words to vectors within the vocabulary through the fastText (Meta) model [66], pretrained with an extensive corpus. Specific pretrained monolingual models were used for each language (cc.en.300.bin for English and cc.es.300.bin for Spanish), both downloaded from the official fastText website. These were trained separately on each language’s Common Crawl and Wikipedia corpora under an identical configuration (Continuous Bag of Words) with position-weights, 300 dimensions, character n-grams (length 5), window size 5, and 10 negatives. Distances between adjacent vectors were stored in a time series. Semantic consistency was determined by calculating the variance of this combined time series. A higher value in semantic consistency is observed in texts where consecutive words represent distant concepts. As in previous NLP research [6], variables were analyzed whenever feasible, considering their mean, SD, skewness, kurtosis, minimum value, and maximal value.

### Data Analysis

Within- and between-language analyses were performed following identical steps (Figure 1D). The within-language analysis used 80% of participants from each group for model training, and the remaining 20% were set aside as a hold-out sample for testing. The between-language analysis used the same training model as the English-language experiment and used the entire Spanish-speaking sample for testing. In all cases, we considered three classification scenarios based on (1) acoustic features only, (2) linguistic features only, and (3) both types of features combined via early fusion (Figure 1E). We trained the model with dedicated speaker-independent training and test splits to ensure robustness. Note that the Spanish-speaking cohort was used exclusively as an independent testing set.

We used a modified algorithm that combines decision trees with a partially connected, sparse multilayer perceptron (MLP) network [80,81]. This network’s structure is based on information from a group of decision trees trained to discriminate AD. It is not constructed with a fully connected layer; the interconnections are guided by the decision tree classifier preceding the MLP. Each neuron specializes in processing a specific tree, taking input from features generated by the decision tree. Importantly, the MLP’s decision tree component improves interpretability, which is important for clinical relevance, and its sparsely connected nature results in fewer parameters, reducing the risk of overfitting. Feature importance is assessed by counting the neurons connected to a specific feature (the more connections, the greater the importance).

The models underwent a bootstrapping strategy, with an 80/20 split. Features were normalized as *z*-scores relative to the train set parameters. To reduce the risk of overfitting, several strategies were implemented during model training [82]. Ridge regularization [83] was applied to penalize large weight magnitudes, encouraging simpler models that generalize better to unseen data. Early stopping [84] was used with a patience of 10 epochs, halting training when validation performance ceased to improve and preventing the model from overadapting to the training data. Within each feature set (speech timing, linguistic, and fusion), we report the best-performing combination of trees ∈ {10,20,40,60,80,100} and depth ∈ {4,6,8,10} (“depth” refers to the number of levels or splits in a single decision tree, indicating its complexity, while “number of trees” refers to the quantity of decision trees used in an ensemble model, such as a random forest or XGBoost [Extreme Gradient Boosting]) across both settings (within-language and between-language), based on the area under the receiver operating characteristic curve (AUC) values. Given the class imbalance problem, we used weighting techniques to assign a higher penalty to misclassification of the minority class, thus adjusting the cross-entropy loss within the neural network [85]. Details about hyperparameter tuning are offered in Multimedia Appendix 1 (Sections S3 and S4). The corresponding code implementations are available on GitHub [86]. Pairwise DeLong tests were conducted to assess whether AUC differences across models were statistically meaningful.

Additionally, we examined whether speech timing and linguistic features in each language were predictive of patients’ MMSE scores (Figure 1F). We report the best correlation across both languages in each case based on the Spearman correlation coefficient (ρ) and the root-mean-squared error (RMSE). This approach indicates how close the predicted values are to the true values on average.

### Ethical Considerations

Data collection procedures of the English dataset (from the publicly available Pitt Corpus) were approved by the Institutional Review Board of the University of Pittsburgh. Participants provided informed consent in accordance with the protocols established by the Alzheimer and Related Dementias Study at the University of Pittsburgh School of Medicine. Data from the Chilean cohort were collected with approval from the Ethics Committee of the Servicio de Salud Metropolitano Oriente, Santiago (project code: ANID-Fondap 15150012). All participants provided written informed consent under the 1964 Declaration of Helsinki. The Spanish dataset’s ethical approval allowed for data to be integrated with international datasets for projects involving the principal investigators (note that these data were analyzed by our local research team rather than shared with external collaborators). The English dataset is open for cross-national analyses, as shown in previous papers and challenges [17,34,43]. Both datasets were deidentified via the removal of personal information (names, occupations, and geographical locations). Data shared in our repository was limited to deidentified feature sets; there being no raw audio recordings (in particular, the speech timing features we targeted cannot be leveraged to infer a speaker’s identity). Importantly, the picture description task we used elicits no self-referential information, as verified manually in each of the transcriptions. Additionally, we ensured harmonization of ethical oversight across both sites by aligning our procedures with international best practices for secondary use of sensitive speech data. Privacy and data protection were guaranteed by (1) robust deidentification, (2) restricting analyses to derived features that cannot be reverse-engineered into intelligible speech or personal identity, and (3) refraining from sharing raw audio between sites. These safeguards are consistent with concerns highlighted in the speech technology and legal communities regarding General Data Protection Regulation compliance and privacy protection [87]. Accordingly, ethical clearance covered the full scope of our cross-linguistic analyses and ensured compliance with data governance requirements at both sites. No compensation was offered or given to participants.

## Results

### Within-Language Results

**Figure 2:**
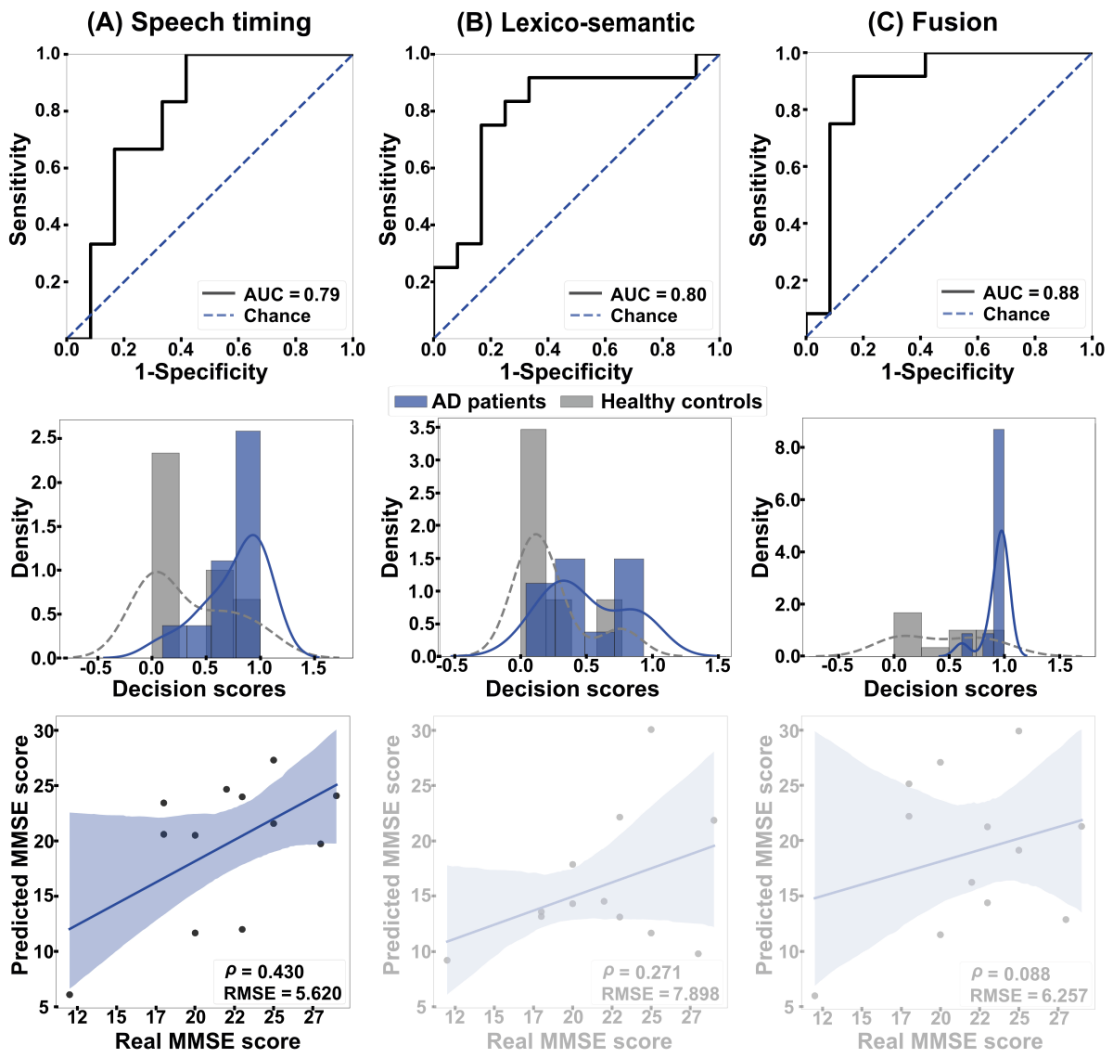
Within-language results. The ROC curves (top row) and the classification scores (middle row) show outcomes for classifying AD patients and HCs in the English-to-English setting. (A) Discrimination was good when based on speech timing and (B) lexico-semantic features alone, and (C) excellent when combining both modalities via early fusion. The bottom row shows correlations between actual and predicted MMSE scores. Associations were significant and moderately strong for all 3 feature sets. AD: Alzheimer disease; AUC: area under the curve; HC: healthy control; MMSE: Mini-Mental State Examination; RMSE: root mean squared error; ROC: receiver operating characteristic curve.

As shown in Figure 2, within-language (English-to-English) analyses revealed similar classification based on timing features from all words and stop words (AUC=0.79, Figure 2A) and based on lexico-semantic features (AUC=0.80, Figure 2B). The fusion of both dimensions yielded maximal discrimination (AUC=0.88, Figure 2C). The outcomes of these 3 classifiers did not differ significantly (speech timing vs lexico-semantic features: *P*=.96; lexico-semantic features vs fusion: *P*=.55; speech timing vs fusion: *P*=.52). Further, the MMSE scores of English-speaking patients correlated significantly with those predicted by within-language regressions based on speech timing features (ρ=0.430, *P*<.001, RMSE=5.620), but not with those based on lexico-semantic features (ρ=0.271, *P*=.39, RMSE=7.898) or the combination of both feature sets (ρ=0.088, *P*=.79, RMSE=6.257).

### Between-Language Results

**Figure 3:**
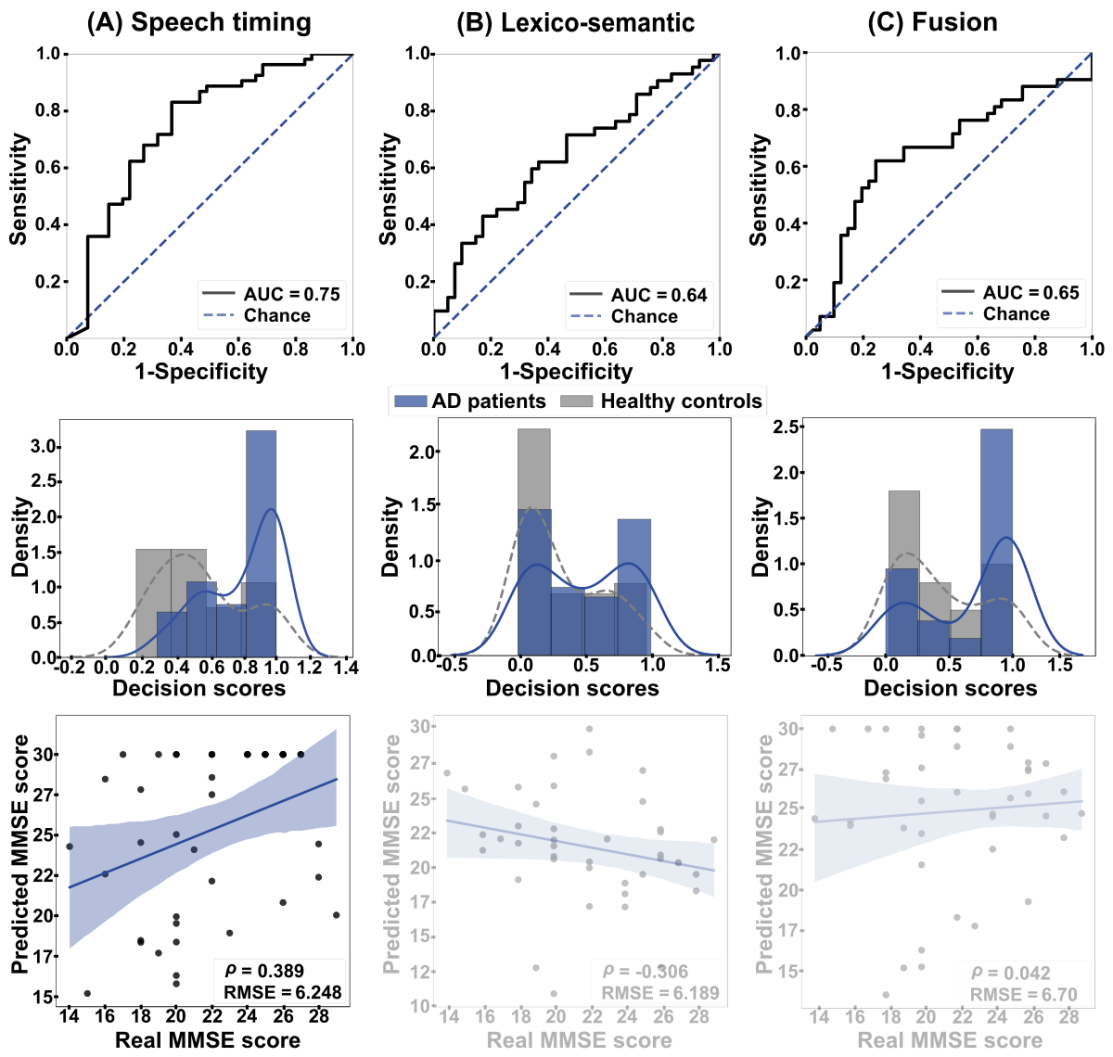
Between-language results. The ROC curves (top row) and the classification scores (middle row) show outcomes for the classification between AD patients and HCs in the English-to-Spanish setting. (A) Discrimination was good when based on speech timing and (B) poor when based on either lexico-semantic features or (C) the combination of both dimensions via early fusion. The bottom row shows correlations between actual and predicted MMSE scores. (A) A strong, significant association emerged when using speech timing features, while (B) nonsignificant correlations were obtained when using lexico-semantic features or (C) the combination of both dimensions. AD: Alzheimer disease; AUC: area under the curve; HC: healthy control; MMSE: Mini-Mental State Examination; RMSE: root mean squared error; ROC: receiver operating characteristic curve.

Between-language (English-to-Spanish) analyses (Figure 3) yielded maximal discrimination for the speech timing classifier, based on stop words (AUC=0.75, Figure 3A). Lower outcomes were obtained for lexico-semantic features, considering semantic information (AUC=0.64, Figure 3B), and the fusion of both dimensions (AUC=0.65, Figure 3C). The speech timing model performed significantly better than lexico-semantic features (*P*=.015) and the fusion of both dimensions (*P*=.005), while the latter 2 models did not differ significantly (*P*=.90). The MMSE scores of Spanish-speaking patients correlated significantly with those predicted by between-language regressions based on speech timing features (ρ=0.389, *P*<.001, RMSE=6.248). Correlations were not significant for regressions based on lexico-semantic features (ρ=–0.306, *P*=.05, RMSE=6.189) or the combination of both feature sets (ρ=0.042, *P*=.79, RMSE=6.470).

To benchmark against commonly used classifiers, we rerun our cross-linguistic analysis using a support vector machine and a support vector regressor. For the support vector machine, the best performance in Spanish was obtained with lexico-semantic features (AUC=0.603), while speech timing and combinations of features performed worse (AUC≤0.458). For the support vector regressor, correlations between predictions and true labels were very low and nonsignificant across feature sets (eg, lexico-semantic features: ρ=–0.075, *P*=.64; speech timing: ρ=–0.008, *P*=.96; combination of features: ρ=–0.131, *P*=.499), with RMSEs ≥4.46.

## Discussion

This study used interpretable ASLA features to identify persons with AD in within- and between-language settings. Trained on data from English speakers, our models detected English-speaking patients through timing and lexico-semantic features (especially when combined), with timing features yielding the best prediction of MMSE scores. Conversely, testing on Spanish speakers showed that only speech timing features were useful for identifying patients and capturing their MMSE scores. These findings inform the quest for clinically relevant and cross-linguistically valid ASLA features, as discussed below.

Within-language classification reached AUC values of 0.79 and 0.80 when based on speech timing and lexico-semantic features, respectively. These classification scores resemble those of other acoustic [17,24,33] and linguistic [18,64,88] studies while meeting the requirements of interpretability. As proposed in previous AD research, longer pauses and syllables likely reflect reduced word retrieval efficiency [89]. Extended silences and phonated segments are typically observed when intended words are not easily retrieved, either because they are not optimally entrenched in memory or because reduced contextual awareness complicates decisions among other lexical candidates. Compatibly, the observed lexico-semantic alterations replicate previous findings from AD research [7,30]. These include high semantic variability and low semantic granularity, pointing to greater variance in conceptual proximity across words and reduced conceptual precision [7,30]. Such patterns also seem absent in other neurodegenerative disorders [7,30], highlighting their role as distinct candidate markers of AD.

Of note, joint analysis of both dimensions yielded an AUC of 0.88, corroborating that within-language AD detection is higher for multimodal (acoustic plus linguistic) than unimodal (eg, linguistic only) ASLA approaches [8,31]. Importantly, these results approximate others derived from uninterpretable audio- and text-level embeddings (eg, Wav2vec and transformer-based models such as BERT) [60,90], suggesting that focus on interpretable ASLA features does not entail a loss of sensitivity. Briefly, the integration of strategic acoustic and linguistic features can optimize AD identification while illuminating patients’ cognitive dysfunctions.

Further, timing features successfully captured patients’ MMSE scores in the within-language setting. This replicates previous works based on timing and other audio-derived features [88,91,92], underscoring the importance of speech rhythm as a candidate marker of the disorder [93]. Specific speech dimensions, then, seem related to overall cognitive status in AD—a promising finding given the vast intra- and interindividual cognitive heterogeneity across patients [94].

Additional insights were obtained through between-language analyses. Identification of AD patients was significantly better for timing features (AUC=0.75) than for lexico-semantic or fusion features (AUCs<0.65). Accordingly, we propose that low word retrieval efficiency (captured by timing metrics) would represent a general cognitive trait of AD, affecting verbal production irrespective of the patient’s language. Indeed, this dimension has enabled AD detection in separate studies targeting languages as diverse as English, Spanish, and Mandarin Chinese [25,95-97]. Conversely, lower cross-linguistic generalization for lexico-semantic patterns would follow from the vast vocabulary differences across typologically distant languages [35,36]. Indeed, specific linguistic patterns may exhibit different and even opposite alterations in AD depending on the patients’ language—for example, pronouns are overused by English-speaking patients and underused by patients who speak Bengali, a language with a more complex pronoun system [16]. Importantly, features yielding moderate AUCs can still support clinical decision-making when interpretability and generalizability are prioritized [98]. Briefly, timing features seem to outperform lexico-semantic features as potential cross-linguistically valid markers of AD.

The poor cross-linguistic performance of lexico-semantic features invites reflection for further research. First, weak classification and MMSE prediction results may reflect the inadequacy of the specific features we used rather than an overarching futility of lexico-semantic measures for between-language studies. In fact, it might even be that these very features do generalize robustly between other (eg, typologically close) language pairs. Further, beyond our study’s goals, cross-linguistic generalizability might be enhanced through alternative strategies, including transfer learning and model training with data from multiple languages [99].

This notion is reinforced by MMSE estimation results. In the between-language setting, MMSE scores were captured by timing features, but not by the lexico-semantic or fusion models. Compatibly, a model trained on silence features and acoustic embeddings from English-speaking patients with AD and HCs yielded good prediction of MMSE scores in a Greek-speaking sample [33]. Thus, speech timing features emerge as promising candidates not only for identifying patients but also for estimating symptom severity across typologically different languages.

Note that our approach cannot ascertain the cause of observed anomalies. In particular, speech timing anomalies may certainly reflect cognitive deficits, but they may also reflect motor speech dysfunction. While motor deficits are rarely reported in AD, they may be present in several cases [100], inviting new studies integrating relevant measures for patient stratification or covariance analysis. The same analytical approaches could be used with strategic measures (eg, picture naming response times) to test the conjecture that speech timing metrics reflect lexical retrieval effort.

Our results bear clinical implications. First, the features used here, as opposed to scores from black-box models, may enhance patient phenotyping and therapeutic decision-making. Indeed, they can be linked to specific cognitive dysfunctions that practitioners are well versed in. Second, they indicate that some, but not all, findings from Anglophone samples may be useful for assessing AD in other Indo-European language communities. The point is important because of the overrepresentation of English in ASLA studies [16,46] and the tendency to derive language-agnostic conclusions from the Pitt Corpus (the English-language dataset used here, which has been leveraged by numerous studies) [17,19,32,44,46] and wide-ranging research challenges [17]. Third, our approach rests entirely on automated methods, increasing cost-efficiency and scalability while minimizing the potential for human error. In this sense, our pipeline has been deployed in a recent version of the Toolkit to Examine Lifelike Language, a speech testing app used in several clinics worldwide [11,101,102]. This allows for its widespread implementation in actual screening, diagnostic, and monitoring scenarios—a key milestone for bridging the gap between basic research and real-world applications.

Yet, our work is not without limitations. First, the 2 datasets we used differed in terms of acquisition conditions. Although we included gold-standard audio normalization steps ensuring comparability, it would be useful to replicate our approach with harmonized cross-linguistic protocols. Second, AD in the English dataset was established before the release of contemporary diagnostic criteria. Although we chose patients with AD-consistent MMSE scores and ensured their sociodemographic matching with same-language controls as well as Spanish-speaking groups, future works should replicate our approach with Anglophone cohorts fulfilling NINCDS-ADRDA criteria or other validated standards. Third, we acknowledge that the limited sample size, especially in the Spanish cohort, restricts statistical power and constrains our capacity to rigorously assess model generalization. Future works should aim for substantially larger groups and include models trained with Spanish speakers and tested on English speakers—thus illuminating their potential for bidirectional generalizability while enabling alternative analytical approaches, based on cross-linguistic calibration or transfer. Fourth, while we controlled for sociodemographic variables, and participants’ dialects were consistent within language groups, our datasets lacked data on education quality, cultural communication norms, and other factors influencing speech production. Future studies should be strategically designed to tackle these factors.

Fifth, we lacked data on when and how examiners intervened during participants’ descriptions. While all examiners’ segments were removed, future works should contemplate the potential impact of this factor. Sixth, our cross-sectional design reveals features that are sensitive to patients’ current cognitive status, but moot on their clinical trajectories. Longitudinal datasets should be leveraged to examine whether these features can predict AD progression. Seventh, though vital for enhancing VAD and downstream processing [7,17], denoising in our preprocessing pipeline might have introduced speech artifacts. These were mitigated through manual revision of all processed audio files and careful tuning of the algorithm’s threshold parameters to minimize unintended distortions. Yet, future studies with more controlled acoustic conditions (eg, in recording booths) could replicate our approach without applying denoising. Eighth, while our 0-shot framework provides insights into the cross-linguistic generalizability of interpretable features, we recognize that performance could likely be improved through few-shot learning strategies, particularly if small amounts of target-language data are available [16,17]. Prior studies leveraging embeddings have demonstrated such gains [32], though often at the cost of transparency. Future research should explore hybrid approaches that incorporate limited target-language samples to enhance model robustness while preserving clinical interpretability.

Finally, note that we aimed to examine whether a model trained in 1 language can generalize to another using interpretable features. Thus, we avoided multilingual large language models or Wav2vec-style embeddings due to their lack of clinical interpretability. Likewise, we did not aim to train a model with interpretable features from both languages, as this would have hindered insights into cross-linguistic generalizability. Such strategies could be leveraged in further research with different goals. Here, evidence that an English-only model can be applied to a lower-resource language helps in understanding which mainstream (English-based) results might hold promise for global speech and language frameworks. Thus, future works should compare our target features with embedding-based features to establish whether interpretability involves a trade-off with sensitivity.

In sum, this study reveals 2 novel empirical patterns for ASLA research on dementia. Interpretable timing and lexico-semantic features support AD detection and cognitive decline estimation among English speakers, but only timing features generalize well from English to Spanish speakers. This suggests that timing features are more language-agnostic than lexico-semantic features. Further work can aid the search for cross-linguistic and language-specific ASLA markers of brain health.

## Appendix

Multimedia Appendix 1 Lists, hyperparameter tuning, and results.

## Abbreviations

AD: Alzheimer disease
ASLA: automated speech and language analysis
AUC: area under the receiver operating characteristic curve
BERT: Bidirectional Encoder Representations from Transformers
CHAT: Codes for the Human Analysis of Transcripts
CHILDES: Child Language Data Exchange System
DSM: Diagnostic and Statistical Manual of Mental Disorders
HC: healthy control
MLP: multilayer perceptron
MMSE: Mini-Mental State Examination
NINCDS-ADRDA: National Institute of Neurological and Communicative Disorders and Stroke and the Alzheimer’s Disease and Related Disorders Association
RMSE: root-mean-squared error
SoX: Sound Exchange
VAD: voice activity detection
XGBoost: Extreme Gradient Boosting

## Data Availability

The demographic information and feature matrices extracted for both datasets are available online [103].
