# Supplementary material for: Automated Speech Markers of Alzheimer Dementia: Test of Cross-Linguistic Generalizability
Source: J Med Internet Res. 2025 Oct 15;27:e74200. doi: 10.2196/74200 (PMC12572752; doi:10.2196/74200)
Supplement: Multimedia Appendix 1 [file jmir_v27i1e74200_app1.docx]

**Multimedia Appendix**

**Section S1.** List of speech timing features.

| **Variable name** | **Operationalization** | **Features**  **for analysis** |
| --- | --- | --- |
| **Whole-recording level** | | |
| Pause duration ratio | Number of pauses / Total audio duration | Ratio |
| Speech segment duration ratio | Number of speech segments/ Total audio duration | Ratio |
| Pause ratio | Number of pauses / Number of speech segments | Ratio |
| Pause duration | Pause duration | Mean, *SD*, skewness, kurtosis min, max |
| Speech segment duration | Speech segment duration | Mean, *SD*, skewness, kurtosis min, max |
| Voiced rate | Voiced rate | -- |
| **Single-word level** | | |
| Word duration | Word duration | Mean, *SD*, skewness, kurtosis min, max |
| Normalized word duration | Word duration / Total audio duration | Mean, *SD*, skewness, kurtosis min, max |
| Word count | Number of words | -- |
| Normalized word count | Number of words / Total audio duration | -- |
| All duration measures are expressed in seconds. | | |

**Section S2.** List of vocabulary selection features.

| **Variable name** | **Operationalization** | **Features**  **for analysis** |
| --- | --- | --- |
| **Word-class features** | | |
| Noun ratio | Number of nouns / Total number of words | Ratio |
| Verb ratio | Number of verbs / Total number of words | Ratio |
| Adjective ratio | Number of adjectives / Total number of words | Ratio |
| Adverb ratio | Number of adverbs / Total number of words | Ratio |
| NNCCW | Number of nouns / Total number of content words | Ratio |
| NVCCW | Number of verbs / Total number of content words | Ratio |
| NADJCCW | Number of adjectives / Total number of content words | Ratio |
| NADVCCW | Number of adverbs / Total number of content words | Ratio |
| **Semantic features** | | |
| Semantic variability | Semantic distance between  adjacent content word vector | SD |
| Granularity | Distance between each word’s node  on WordNet and the node ‘entity’ | Mean, *SD*, skewness, kurtosis min, max, segmented mean |

NNCCW: Number of nouns over count of content words. NVCCW: Number of verbs over count of content words. NADJCCW: Number of adjectives over count of content words. NADVCCW: Number of adverbs over count of content words.

**Section S3.** Hyperparameter tuning for classification analyses.

| **Hyperparameter** | **Range** |
| --- | --- |
| **n_estimators** | {2, 4, 6, 8, 10} |
| **max_depth** | {20, 40, 60, 80,100} |
| **Learning rate**  **(Adam Optimizer)** | 1e-3 |

**Section S4.** Hyperparameter tuning for regression analyses.

| **Hyperparameter** | **Range** |
| --- | --- |
| **n_estimators** | {2, 4, 6, 8, 10} |
| **max_depth** | {20, 40, 60, 80,100} |
| **Learning rate (Adam Optimizer)** | 1e-4 |

**Section S5.** Supplementary results for the within-language setting.

|  | **AUC** | **UAR** | **Sensitivity** | **Specificity** |
| --- | --- | --- | --- | --- |
| **Speech timing** | 0.79 | 0.71 | 0.83 | 0.58 |
| **Vocabulary selection** | 0.80 | 0.75 | 0.67 | 0.83 |
| **Fusion** | 0.88 | 0.75 | 1.00 | 0.50 |

**Section S6.** Supplementary results for the between-language setting.

|  | **AUC** | **UAR** | **Sensitivity** | **Specificity** |
| --- | --- | --- | --- | --- |
| **Speech timing** | 0.75 | 0.66 | 0.89 | 0.44 |
| **Vocabulary selection** | 0.64 | 0.58 | 0.49 | 0.67 |
| **Fusion** | 0.67 | 0.65 | 0.63 | 0.67 |
